# The contribution of large genomic rearrangements in *BRCA1* and *BRCA2* to South African familial breast cancer

**DOI:** 10.1186/s12885-020-06917-y

**Published:** 2020-05-06

**Authors:** Nerina C. van der Merwe, Jaco Oosthuizen, Magdalena Theron, George Chong, William D. Foulkes

**Affiliations:** 1 Division of Human Genetics, Faculty of Health Sciences, University of the Free State, Bloemfontein, South Africa; 2 Division of Human Genetics, National Health Laboratory Services, Universitas Academic Hospital, Bloemfontein, South Africa; 3 Lady Davis Institute and Segal Cancer Centre, Jewish General Hospital, Montréal, QC Canada; 4 Research Institute of the McGill University Health Centre, Montréal, QC Canada; 5 Program in Cancer Genetics, Departments of Oncology and Human Genetics, McGill University, Montréal, QC Canada

**Keywords:** *BRCA1/2*, Familial breast cancer, Large genomic rearrangements, South Africa, Whole gene deletions

## Abstract

**Background:**

Pathogenic variants that occur in the familial breast cancer genes (*BRCA1/2*) lead to truncated ineffective proteins in the majority of cases. These variants are mostly represented by small deletions/insertions, nonsense- and splice-site variants, although some larger pathogenic rearrangements occur. Currently, their contribution to familial breast cancer (BC) and ovarian cancer (OVC) in South Africa (SA) is unknown.

**Methods:**

Seven hundred and forty-four patients affected with BC or OVC were screened for larger genomic rearrangements (LGRs) by means of multiplex ligation-dependent probe amplification or Next Generation Sequencing using the Oncomine™ *BRCA* research assay.

**Results:**

The patients represented mostly medium to high-risk families, but also included lower risk patients without a family history of the disease, diagnosed at an early age of onset (< 40 years). Eight LGRs were detected (1.1%); seven in *BRCA1* with a single whole gene deletion (WGD) detected for *BRCA2*. These eight LGRs accounted for 8.7% of the 92 *BRCA1/2* pathogenic variants identified in the 744 cases. The pathogenic LGRs ranged from WGDs to the duplication of a single exon.

**Conclusions:**

Larger rearrangements in *BRCA1/2* contributed to the overall mutational burden of familial BC and OVC in SA. Almost a quarter of all pathogenic variants in *BRCA1* were LGRs (7/30, 23%). The spectrum observed included two WGDs, one each for *BRCA1* and *BRCA2*.

## Background

The cumulative risk of developing breast cancer (BC) to the age of 80 years for heterozygotes of *BRCA1* and *BRCA2* pathogenic variants (hereafter, heterozygotes), has been approximated at 72% (95% CI 65–79%) and 69% (95% CI 61–77%), respectively. The risk for developing ovarian cancer (OVC) is lower, at around 44% (95% CI 36–53%) for *BRCA1* and 17% (95% CI 11–25%) for *BRCA2* heterozygotes [1]. Current risk-reducing strategies for BC in heterozygotes include prophylactic surgery to remove the breasts and/or ovaries, increased surveillance with more frequent mammograms along with magnetic resonance imaging starting at a younger age, and risk-reducing medications [2].

South Africa (SA), similar to the rest of the world, is experiencing an increase in the demand for comprehensive *BRCA1/2* testing, due to mainly two factors. These include heightened public awareness after the Angelina Jolie revelations [3], which emphasised the impact and consequences of being a heterozygote, together with the prophylactic management options available. The second contributing factor is that targeted genotyping used for many years for the identification of founder and recurrent SA pathogenic variants have since been proven to be effective only for the Afrikaner and Black isiXhosa populations [4, 5]. The genetic architecture of the various SA population groups required a new approach and resulted in more patients being screened comprehensively [6–11].

Next Generation Sequencing (NGS) was implemented as a more rapid and cost-effective comprehensive screening strategy [7]. Transitioning to this technology, however, was challenging for the diagnostic platform and various validations were performed to prove sensitivity, specificity, and repeatability, especially with regard to the detection of larger genomic rearrangements (LGRs).

Although various SA studies reported comprehensive *BRCA1/2* screening results, to date the contribution of LGRs to familial BC and OVC for the broader SA population has not been determined, apart from a pilot study performed in 2011 by Sluiter and Van Rensburg [12]. They identified a single LGR in a SA Greek patient, and indicated a contribution of 3% (single patient) in a mostly Afrikaner (*n* = 36) and European heritage cohort. We aimed to determine the contribution of LGRs to the *BRCA1/2* mutation spectrum observed in SA familial BC and OVC for the country as a whole. The patients included in this study represented each of the main population groups, namely Black, SA Indian, Coloured and Whites (Afrikaner and non-Afrikaner). In the SA context, patients who self-identified themselves as Coloured, have a complex history of ancestrally derived admixture with the Khoesan, Bantu-speakers, Europeans, and populations from the Indian sub-continent [13], and are regarded as being of mixed ancestry.

## Methods

The study was approved by the Ethics Committee of the Faculty of Health Sciences at the University of the Free State in Bloemfontein (ETOVS 31/95, ETOVS 65/08, ECUFS 107/2014 and ECUFS 108/2014). Permission was also obtained from the National Health Laboratory Services for the use of the data.

Seven hundred and forty-four BC and/or OVC patients (including 129 patients described by Moeti [14]) attending various genetic clinics were received for comprehensive screening of *BRCA1/2*. All patients underwent pre- and post-test counselling at their respective referring hospitals during which they provided information about their personal and familial history and gave written informed consent for genetic analysis.

The patients represented medium (two related family members affected with the disease, *n* = 415) to high-risk families (minimum of three related affected family members, *n* = 134), but also included low familial risk patients (with no family history of breast and/or OVC, *n* = 195) who were diagnosed at an early age of onset (< 40 years). Each request included a family pedigree (if applicable) and clinical details of the pathology. Documents pertaining to patients’ informed consent are stored at the respective referring hospitals. Population group was determined by patient self-identification. The cohort included 277 Black (37.2%), 140 SA Indian (18.8%), 85 White non-Afrikaner (11.4%), 110 White Afrikaner (14.8%) and 132 Coloured (17.7%) patients.

Genomic DNA was isolated from whole blood using the salting-out method [15]. For high-resolution melting analysis (HRMA), the quality and quantity of DNA samples were assessed with the NanoDrop® ND-100 Spectrophotometer v3.01 (NanoDrop® Technologies Inc., Wilmington, DE, USA), whereas the Qubit dsDNA High Sensitivity assay kit was used to quantify DNA with the Qubit® Fluorometer (Invitrogen; Thermo Fisher Scientific, Inc., Waltham, MA, USA) for NGS. Reference sequences used for *BRCA1* and *BRCA2* analyses were GenBank NM_007294.3 (*BRCA1*) and NM_000059.3 (*BRCA2*).

Conventional mutational analysis for single nucleotide variants (SNVs) and smaller indels was initially performed for a subset of BC patients from these clinics, as described previously [6, 8]. This approach entailed a combination of HRMA, the protein truncation test, and Sanger sequencing. NGS was performed for the remainder of samples (*n* = 615) by means of the Oncomine™ BRCA Research Assay (Life Technologies, Carlsbad, CA, USA). The primer pools targeted the entire coding region including small areas of intronic flanking sequences for both genes. Multiplexed primer pools were used to construct the amplicon library using PCR-based targeted amplification. Sequencing was performed on the Ion Proton Platform (Life Technologies, Carlsbad, CA, USA).

The Ion Reporter™ Software (Life Technologies, Carlsbad, CA, USA) was used to filter out possible artifacts. Raw signal data were analysed using the Torrent Suite™ versions 5.2, 5.4, 5.6, 5.10 and 5.12. The pipeline included signalling processing, base calling, quality score assignment, trimming of the adapters (average read length 114 bps), read alignment to and quality control of mapping quality. Coverage analysis and variant calling was generated using the Torrent Variant Caller plugin software in the Torrent Server. The average coverage depths obtained were 489X (range 151–1893X).

Copy number variation (CNV) detection was performed using an algorithm based on the normalisation of read coverage across amplicons to predict the copy number or ploidy states. Read coverage was corrected for guanine (GC) bias prior to copy number state determination and compared to a baseline coverage that was constructed using a minimum of 60 control samples (each with an average of 24 million bases called and a read count of 215,000), using regions with known ploidy states (https://assets.thermofisher.com/TFS-Assets/LSG/brochures/CNV-Detection-by-Ion.pdf). CNVs were confirmed using multiplex ligation-dependent probe amplification (MLPA).

Patients screened by means of the conventional techniques were also subjected to the analysis for LGRs using MLPA. MLPA was performed using the SALSA® MLPA® P002-C1 and SALSA® MLPA® P002-D1 for *BRCA1*, with SALSA® MLPA® P045-B3 used for *BRCA2* (MRC-Holland, Amsterdam, The Netherlands). The ligated products were run together with a size standard on an ABI 3130XL Genetic analyser (Applied Biosystems, Carlsbad, California, USA). MLPA-positive results were corroborated using the confirmation assays SALSA® MLPA® P087-C1 for *BRCA1* and SALSA® MLPA® P077-A3 for *BRCA2*. MLPA data were analysed using GeneMarker® software version 2.6.4 (SoftGenetics, LCC, State College, PA, USA). The CNVs were named according to the Human Genome Variation Society (http://www.HGVS.org/varnomen) guidelines and classified using the adapted recommendations of the American Society of Medical Genetics and Genomics (ACMG) for the interpretation and reporting of single-gene copy number variants [16].

Genotype analysis was carried out for 21 individuals representing the family of patient 13/08, using the D17S250, D17S579 and D17S855 markers for *BRCA1* [10]. Forward primers were end-labelled with ^32^P in a 10 μl reaction before conventionally amplified in 20 μl reactions. The samples were diluted 2:1 with a loading dye (95% formamide; 12.5 mM EDTA, pH 8, 0.05% bromophenol blue and 0.05% xylene cyanol), denatured for 5 min at 95 °C and 5 μl loaded onto a 6% denaturing polyacrylamide gel, together with a sequencing ladder.

## Results

Of the 744 BC and OVC patients, 92 patients (12.3%) carried a pathogenic *BRCA1/2* variant (*BRCA1* 30/744; 4.0% and *BRCA2* 62/744; 8.3%). The higher prevalence for *BRCA2* was driven by the presence of two founder mutations present in the Afrikaner (*BRCA2* c.7934del,p.Arg2645AsnfsX3, historically known as *BRCA2* 8162delG) and Black (*BRCA2* c.5771_5774del,p.Ile1924ArgfsX38, historically known as *BRCA2* 5999del4) populations. All 744 cases were screened for the presence of LGRs. Overall, 8/92 *BRCA1/2* mutated cases had an LGR (8.7%), with *BRCA1* contributing more LGRs (7/30, 23.3%) compared to *BRCA2* (1/62, 1.6%) (*P* = 0.0014 for the difference, Fisher’s exact test). Eight different LGRs were identified, seven in *BRCA1* detected by NGS, and one in *BRCA2* using MLPA only. All eight LGRs were confirmed by additional confirmation MLPA assays. Six of the LGRs represented various smaller intragenic exon microdeletions/duplications (6/8, 75.0%), with two whole gene deletions (WGDs) detected (2/8, 25.0%).

LGRs were detected in 1.1% (8/744) of the study population and accounted for 8.7% (8/92) of all the positive results obtained. The majority of CNVs was observed for the non-Afrikaner White population (3/85, 3.5%), followed by 1.4% for both the SA Indian (2/140) and Coloured (1/132) populations respectively. The Black and White Afrikaner groups had the least amount of CNVs, with 0.7% positives identified for the Black patients (2/277) and an absence of CNVs among the Afrikaner (0/110; 0%). Seven of the index patients presented with BC, whereas the eighth presented with OVC. The age at onset of the disease ranged from 32 to 48 years, with a mean age of 38.9 years. Six of the eight patients reported a family history of BC and other malignancies, whereas the patients carrying a *BRCA1* exon 21 deletion and a complete *BRCA2* deletion were not aware of any cancers in the family.

**Fig. 1:**
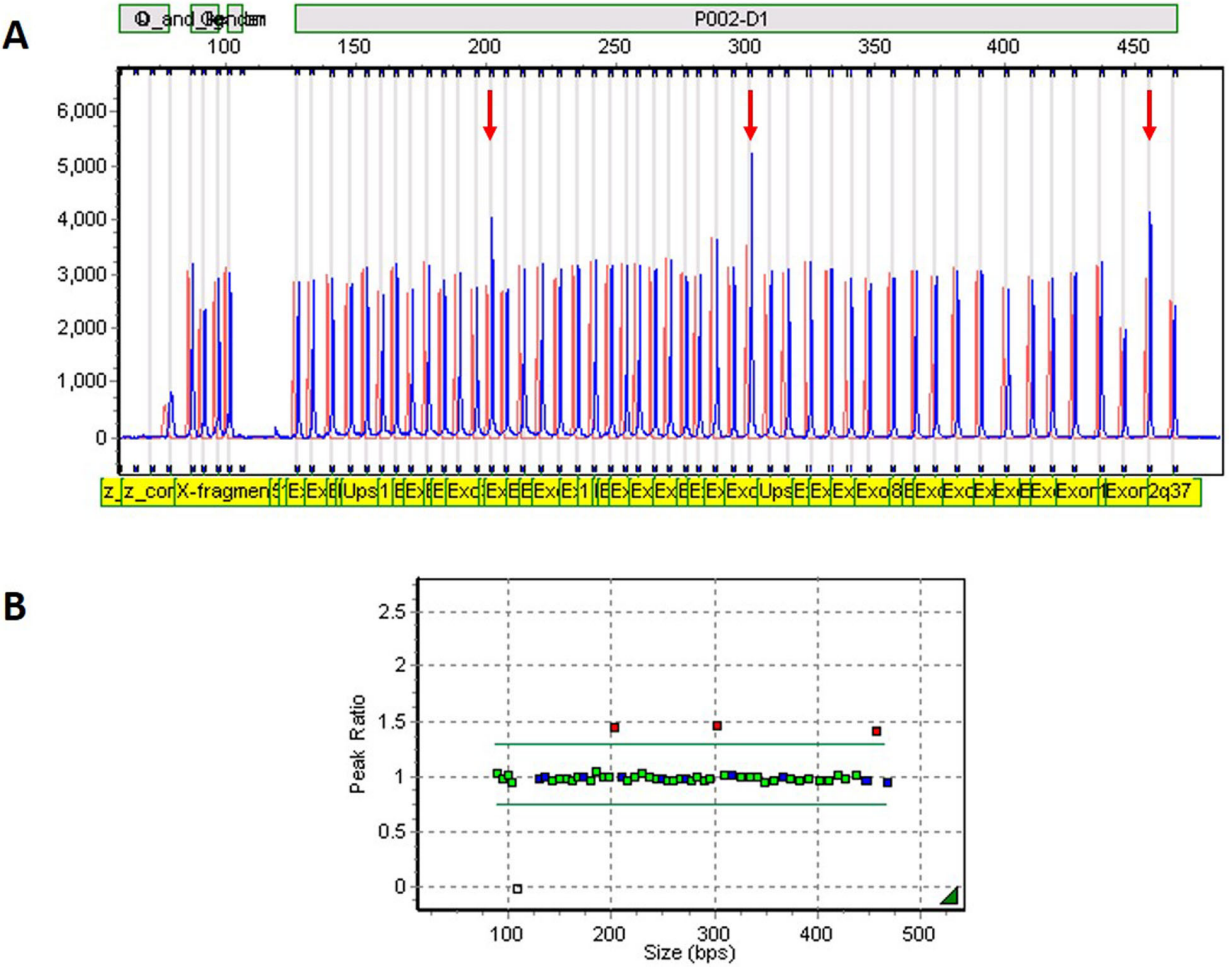
Confirmation of the presence of a 6-kb duplication of exon 12 (formerly known as exon 13) detected for index 1220/15 in *BRCA1* using the SALSA® MLPA® P002-D1 probe mix. **a**. Raw data indicating a duplication of three probes (sized 202, 301 and 459 kb). **b**. Graphical representation of the results using GeneMarker® software from SoftGenetics

A duplication of *BRCA1* exon 12 [formerly exon 13; NG_005905.2(LRG_292):g.(141369_141497)dup] was observed for a single White non-Afrikaner patient (patient 1220/15). The duplication was indicated for all three probes representing exon 12 (Fig. 1) listed in the MLPA product description version D1–02 (issued 17 September 2015). The duplication was detected for a BC patient diagnosed with premenopausal ductal carcinoma (T3N2M0) who reported two first-degree relatives affected with early-onset disease.

The second LGR detected involved the deletion of *BRCA1* exons 1a, 1b and 2 [NG_005905.2(LRG_292):g.(?_ 93,968)del] observed for an African patient from Zimbabwe (2074/18) and a White non-Afrikaner patient (13/08). The deletion was initially detected by NGS and confirmed by MLPA using the *BRCA1* P002-D1 probe mix (data not shown). The deletion was evident from five probes, indicating the presence of a single copy of the region (data not shown). Patient 2074/18 was diagnosed with triple-negative unilateral BC in her thirties. She reported a first-degree relative affected with skin- and OVC at an early age (≤ 45 years).

Patient 13/08 was diagnosed with early-onset OVC (≤ 45 years). The right ovary contained a large cystic tumour, with a smaller tumour on the left. The histological features were representative of a moderately to poorly differentiated carcinoma. The family history entailed three first-degree relatives affected with BC. Genotyping of family members at three short tandem repeat markers in and around *BRCA1* indicated a common haplotype co-segregating with the variant (family tree not illustrated).

A deletion of exons 4 to 6 of *BRCA1* [NG_005905.2(LRG_292):g.(111450_113863)del] was observed for a single White non-Afrikaner patient (1884/18) (data not shown). The deletion was detected by NGS and confirmed using the MLPA *BRCA1* P087-C1 kit. This LGR was detected for a BC patient diagnosed with invasive triple-negative ductal carcinoma in her forties. The patient reported a first-degree family member diagnosed with early-onset OVC, who passed away within 5 years of diagnosis. The maternal history also included two distant family members affected with BC. The patient was of English and Irish descent.

Another deletion involving *BRCA1* exon 17 [NG_005905.2(LRG_292):g.(154032_154111)del] was observed for an SA Indian patient affected with unilateral BC at an early age (≤ 40 years). The deletion was initially detected by NGS. The deletion involved a single exon and therefore the result was confirmed using the *BRCA1* P002-D1 probe mix (data not shown). The patient presented with invasive ductal carcinoma (ER-, PR+ and HER2-). The family history comprised three distant female relatives affected with an unknown cancer, BC (diagnosed late) and a diagnosis of throat cancer, respectively.

Exon 21 of *BRCA1* [NG_005905.2(LRG_292):g.(168789_168864)del] was deleted in an African female patient diagnosed with early-onset BC (≤ 40 years). As the deletion involved a single exon, the deletion was confirmed using an alternative probe mix (namely *BRCA1* P087-C1) to exclude a false positive result due to the presence of polymorphisms in the binding and ligation regions of the probes (data not shown). This patient reported no family history of cancer. Unfortunately, no tumour characteristics were indicated.

**Fig. 2:**
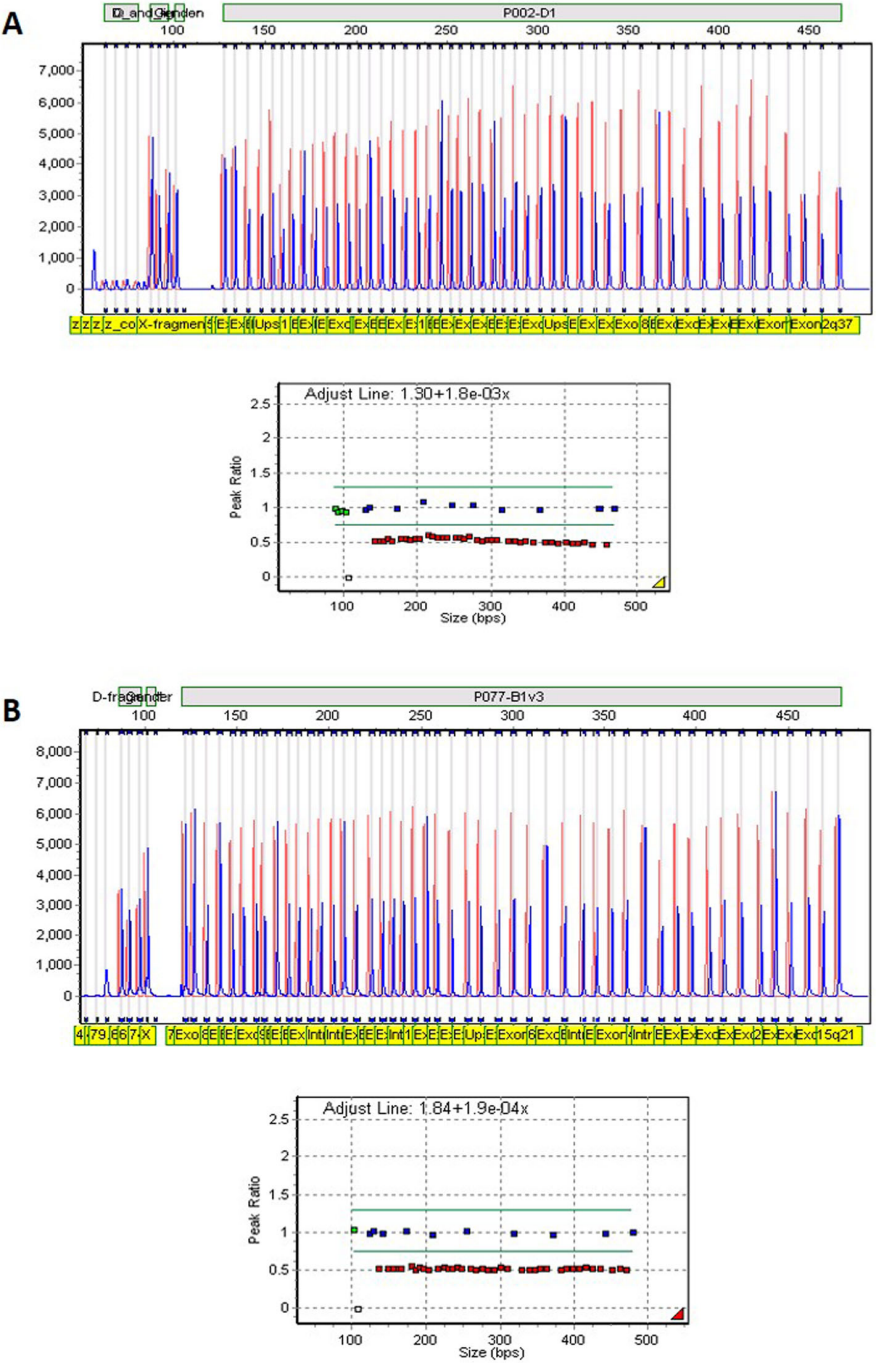
Presence of a complete deletion involving *BRCA1* (index 1428/16) and *BRCA2* (index 1305/16), respectively. **a**. Raw data and graphical MLPA presentation of results for index 1428/16 using GeneMarker® software for SALSA® MLPA® P087-C1 indicating a single copy for all probes representing *BRCA1*. **b**. Raw data and graphical presentation of MLPA results for index 1305/16 for SALSA® MLPA® P077-A3 indicating a single copy for all probes representing *BRCA2* (indicated in red)

A complete deletion of the entire *BRCA1* gene [NG_005905.2(LRG_292):g.(93887_172308)del] was observed for a Coloured woman. The index (1428/16) was diagnosed with BC at a very young age (≤ 35 years) and had an extensive family history of breast and other cancer types. The deletion was detected using the *BRCA1* P002-D1 kit and was confirmed by the *BRCA1* P087-C1 probe mix (Fig. 2a). Segregation of this variant could not be confirmed, as no other affected family members have been tested thus far. The breakpoints of this deletion were not characterised. The deletion did, however, include an upstream region encompassing *NBR2* (data not shown).

The complete deletion of *BRCA2* [NG_012772.3(LRG_293):g(5982_882910)del] was observed for an SA Indian female (1305/16), diagnosed with premenopausal triple-negative ductal BC (Fig. 2b). As the patient did not report any cancer in the family, the pathogenic variant was regarded as de novo. The deletion was detected using the *BRCA2* P045-B3 kit and confirmed with *BRCA2* P077-A3 probe mix. The index preferred not to be involved in further investigations. Therefore, no segregation analysis could be performed. Although the breakpoints of this whole gene deletion were not characterised, the results indicated a minimum size of 104 kb. The P045-B3 kit indicated not only heterozygosity for *BRCA2* (~ 84 kb), but also for an area 20 kb upstream of *FRY* in exon 61 and included the small *ZAR1L* and *RP11-37E23.5* genes situated in-between (data not shown).

The presence of SNVs resulted in the detection of false positive CNV findings in 0.9% (7/744) of the cohort during routine use of the *BRCA1* P002-D1 and *BRCA2* P045-B3 probe mixes. The percentage of false positive results was increased due to one of these SNVs representing the Afrikaner founder pathogenic variant located in *BRCA2* exon 17 [17]. The position of these SNVs influenced the binding and ligation of the probes (data not shown). These false positive findings were not observed in any of the confirmation kits used, although these probe mixes have not been used as extensively as P002-D1 and P045-B3.

## Discussion

The eight gene variants involving LGRs identified for SA BC and OVC patients, cover the entire range of possible CNV types, as they include two different WGDs to patients with single intragenic exon deletions or duplications. According to the latest amendments of the ACMG guidelines applicable to CNVs, these variants were classified using various parameters [16]. As the two WGDs affect all known coding exons involved in *BRCA1* and *BRCA2* where loss of function is the definitive mechanism of disease, they were classified as pathogenic Class 5 based on PVS1 alone. The two multi-exon CNVs involving exons 1a–2 and exons 4–6 deletion, each includes a critical domain, namely the initiation site for protein translation Met1 and the RING finger binding domain, which is required for specific hetero-complex formation between BRCA1 and BARD1 [18]. As both these regions include regions critical to protein function, they were also classified as Class 5 pathogenic variants based on PVS1.

Two of the CNVs identified each represent the deletion of a single exon, namely *BRCA1* exon 17 and *BRCA1* exon 21. According to the new amended guidelines, these have to be interpreted with care with regard to their pathogenicity to prevent incorrect classification [16]. Both these exons form part of the C-terminal BRCT repeat domain (aa1663–1866), which mediates protein-protein interactions [19]. As the deletion of the respective exons is not in frame, it will result in nonsense-mediated decay of the altered transcript. BRCA1 will therefore not co-localise in the nuclear foci with BARD1 and BACH1 [18], preventing DNA repair. Based on these factors, the two variants are characterized as pathogenic Class 5 using PVS1.

The final CNV represents the gross duplication of exon 12 formerly known as exon 13 (ins6kbEx13) in literature. This uncharacterised duplication is likely in tandem and therefore might result in an altered transcript [19]. This transcript will also be subjected to nonsense-mediated decay. These factors resulted in a classification of pathogenic using PSV1.

The 6-kb duplication of *BRCA1* exon 12 detected once in this study represents a founder pathogenic variant in geographically diverse populations such as Great Britain, Canada and Sweden [20, 21]. Haplotype analyses of multiple heterozygous families confirmed a common ancestor for this pathogenic variant, which most probably originated in the northern regions of Great Britain. The authors proposed screening for this deleterious variant in countries with historical links with Britain (such as SA) and proved to be correct, as the SA heterozygote 1220/15 reported a British/Norwegian heritage.

Deletions involving *BRCA1* exons 1a, 1b and 2 have been documented frequently [7, 22–30] and show a strong association especially with the Latin American/Caribbean ancestry [31]. The deletions either occur due to the presence of a large duplicated region (ψ*BRCA1)* upstream of *BRCA1* [23], or due to homologous recombination between multiple Alu elements present in both *BRCA1* and the pseudogene [22, 32]. This region upstream of *BRCA1* creates a hot spot for unequal recombination, resulting in LRGs [23].

Six different breakpoints have been reported before, with deletions ranging in size from 8 kb to ~ 37 kb pathogenic alleles [12]. Although the deletions started in different regions, the majority all included a section of intron 2 of ψ*BRCA1* and encompassed both *NRB2* and exons 1a, 1b and 2 of *BRCA1* [27]. All the deletions ended in intron 2 of *BRCA1*. The 56-bp fragment located between nucleotides 40,228 and 40,083 (reference sequence AC060780) acting as a bi-directional promoter for *BRCA1* and *NBR2*, were reported to be absent in all the LGRs reported for this region [33], suggesting that no *BRCA1* RNA transcript would be produced [27].

Thus far, two SA deletions involving *BRCA1* exons 1a, 1b and 2 were detected for White BC patients (current study and [7]). As the second patient in which this variant was identified during the current study was Zimbabwean, she was excluded from the statistics calculated for SA. The breakpoints of the deletions were not investigated as the homology between the pseudogene and *BRCA1* makes the region difficult to investigate [34, 35]. The White non-Afrikaner index patient (13/08) indicated a German heritage. Engert et al. [29] reported deletions involving *BRCA1* exons 1a, 1b and 2 in four German BC families. These deletions most probably occurred due to homologous recombination between Alu elements and a stretch from the pseudogene [29].

The deletion of exons 4–6 of *BRCA1* (in the literature also referred to as *BRCA1* del exons 5–7) detected for a single White non-Afrikaner BC patient is rare, as it has been detected only six times previously, mostly in European countries, namely Germany [25], Croatia [36], Italy [37], Slovenia [38], Spain [39] and Denmark [40]. For some of these deletions, the breakpoints were determined [25, 39, 40]. Preisler-Adams et al. [25] determined that a homologous region of 15 bp between *AluSx* in intron 3 and *AluSc* in intron 7 at the crossover site, is responsible for this LGR in German families. The size of the deletion, however, differs for the various countries, as it ranges from 4995 bp to 5024 bp. The size of the SA deletion has not yet been determined.

Exon 17 of *BRCA1* is to date the most frequent single exon involved in larger rearrangements. The deletion of this single exon has been reported for multiple populations, such as the Americans [41], Italians [42], the Irish and Swedish [43], but very specifically for German families [25, 29, 44]. Various studies representing German breast and ovarian cancer families have identified a total of three different large rearrangements involving exon 17 only, namely a 5.1 kb recurrent deletion [44], a founder pathogenic 3.1 kb deletion and a novel smaller deletion with different breakpoints [29]. Together these rearrangements, including those identified involving exons 12 and 22, account for more than 50% of all deletions/duplications found thus far within the German population [29].

This exon deletion was identified for a single SA Indian BC patient. As the deletion of exon 17 has not yet been described for the Indian population of mainland India [45, 46], this pathogenic CNV represents a novel variant specific to the SA Indian population.

The exon 21 deletion detected for the African BC patient is novel, as the deletion of this single exon has not been described before. It has previously always been described as part of larger rearrangements such as exons 20–22 [31, 47]; 20–21 [31]; 21–22 [31, 48, 49]; 21–23 [31, 47]; or 21–24 [31, 50]. This pathogenic variant represents the first to be identified in the SA Black population. Family follow-up studies will be performed to identify at-risk related family members.

The sixth LGR detected in SA represented a rare complete deletion of *BRCA1*. Only a limited number has been reported before for two Galician patients [28], a single American patient [30] and 17 (0.01%) of 48,456 patients representing various nationalities. These nationalities included patients of Latin American/Carribean descent [31], three Spanish patients [51–53] and a Slovakian patient [54]. The majority of these pathogenic variants segregated in families, with only two reported as being de novo [52, 53]. According to the data released by Myriad Genetics [31], there are differences between ancestries in the prevalence of this LGR. Seventeen patients with a complete *BRCA1* deletion were reported, of which 13 originated in Latin America or the Caribbean [31]. An additional patient reported by Jackson et al. [30] was from Mexico. The finding of the current study represents the first report of a complete *BRCA1* deletion for a SA patient.

Breakpoints were determined for two of the previously reported complete gene deletions using single nucleotide polymorphism (SNP) array analyses and revealed a size difference [28, 53]. For the Spanish de novo pathogenic variant, the deletion started from the region surrounding the VAT1 (MIM#604631) locus to the beginning of *NBR1* (MIM#166945). The deletion included *RND2* (MIM#601555), the pseudogene (ψ*BRCA1*), *BRCA1* (MIM#113705) and the *NBR2* complete genes [40]. For the two Galician patients [28], the region encompassed *NBR2* and *BRCA1* only, similar to that tentatively indicated for the SA pathogenic variant. Sequencing of the junction region revealed a smaller region in which two of the five Alu elements located in the breakpoint regions, shared a 20 bp sequence. The authors postulated the size of the deletion to be 109,824 bp (NG_005905.2:g.70536_180359del), which originated due to unequal homologous recombination [28].

Whole gene deletions of *BRCA2* are exceptionally rare. Only four cases have been reported in the literature, of which one was recorded for somatic tissue [55]. This LGR was described for three BC patients, one French male patient with a family history of breast and pancreatic cancer [56], and two female BC patients from Italy [57] and the USA [31], respectively. The female patients represented high-risk patients. Tournier et al. [56] mapped the deletion and concluded that it extended over a minimum of 298 kb. However, the deletion did include several loci corresponding to putative transcripts of unknown functional significance, similar to the SA deletion (data not shown). No information regarding familial segregation existed for any of the cases, including the SA deletion. We speculate that this deletion of *BRCA2* was de novo, as this SA Indian BC patient did not report a family history of cancer.

The present SA study identified seven (eight including the Zimbabwean patient) LGRs, three in non-Afrikaner White patients, two representing the SA Indian population, and one each for the Black and Coloured populations. Taking all previous SA studies listed in Table 1 into account, 1081 BC and/or OVC SA patients representing various ethnicities have been screened thus far for the presence of LGRs in *BRCA1*/*2* (Table 1, excluding the Dutch immigrant reported by Reeves [58] and the Zimbabwean patient screened during of the present study). The current study is therefore the most comprehensive attempt to identify LGRs in a broad group of SA populations. Overall we found that 8.7% (8/92) of *BRCA1/2* pathogenic variants were LGRs. Their contribution to the mutational spectrum is greater than that reported by Sluiter and Van Rensburg [12] (8.7% compared to ~ 3%).

**Table 1:** South African studies that investigated the prevalence of large genomic rearrangements in *BRCA1* and *BRCA2* for the various population groups

Study	Number of patients (percentage with positive results)	Population group	Detection method	Genes	Results
Reeves et al. [10]	90 (0.0)	All	Long range PCR for Dutch founders only	*BRCA1* exon 13 del (IVS12-1643del3835) *BRCA1* exon 22 (IVS21-36del510)	None detected
	60 (0.0)	Afrikaner			
	11 (0.0)	Jewish			
	19 (0.0)	British & European			
Reeves [58]	56 (1.8)	All	MLPA	*BRCA1* only	*BRCA1* exon 13 del in a Dutch immigrant
	55 (0.0)	White			
	1 (100)	Dutch immigrant			
Sluiter and Van Rensburg [12]	52 (1.9)	All	MLPA	*BRCA1* and *BRCA2*	*BRCA1* exons 23–24 del in a SA Greek patient
	36 (0.0)	Afrikaner			
	16 (6.3)	Greek & other			
Francies et al. [7]	108 (0.9)	All	NGS		*BRCA1* 1a-2 del in a White patient
	85 (0.0)	Black			
	2 (0.0)	Coloured			
	16 (6.3)	White			
	5 (0.0)	SA Indian			
Chen [59]	33 (0.0)	Black	MLPA	*BRCA1* and *BRCA2*	None detected
Current study	744 (0.9)	All	MLPA	*BRCA1* and *BRCA2*	Eight LGRs detected: complete *BRCA2* deletion in a SA Indian patient; complete *BRCA1* deletion in a Coloured patient; *BRCA1* 1a-2 del in a White non-Afrikaner and Zimbabwean patient; *BRCA1* exon 12 dup in a White non-Afrikaner patient; *BRCA1* 4–6 del in a White non-Afrikaner patient; BRCA1 exon 17 del in a SA Indian patient; BRCA1 exon 21 del in a Black patient
	277 (0.4)	Black			
	140 (1.4)	SA Indian			
	132 (0.8)	Coloured			
	85 (3.5) 110 (0.0)	White non-Afrikaner Afrikaner			

The largest single published series of BC and OVC patients screened for the presence of CNVs is the Myriad data set (total of 48,456 patients screened) [31]. Here the authors reported an average LGR rate of 7.9% (9.9% for the high-risk group versus the 5.9% for the elective group), of which 90% were observed in *BRCA1*. In another large European series described by Smith et al. [60], the total CNV rate for *BRCA1* and *BRCA2* was slightly higher, namely 17.5% (104/591 families) and 6.2% (34/552 families) respectively, with an average of 11.9%. The results of the current SA study correspond not only to both the American (7.9%) and European (average of 11.9%) rates regarding the contribution of LGRs to familial BC and OVC, but also the mutation range (single intronic gene deletions or duplications to WGDs).

The results of the present study concur with existing knowledge in literature that more LGRs are reported for *BRCA1* compared to *BRCA2* [12, 29]. The increased number of CNVs in this gene is due to the abundance of intronic Alu repeat sequences [61]. These Alu repeats are most probably responsible for unequal homologous recombination and represents one of the most common mechanisms for the creation of CNVs in these genes.

For White Afrikaners, the absence (0%) of LRGs is in stark contrast to the approximately 33% intra-exonic pathogenic variants recorded for this group [5]. This high percentage, however, could be attributed to the presence of a major *BRCA2* Afrikaner founder pathogenic allele [16]. The absence of CNVs in the White Afrikaner population with its European heritage is surprising, as many CNVs (including a founder variant reported for the Netherlands [62]) have been reported for this region of the world. Investigators genotyped a large subset (*n* = 7746) of Afrikaner individuals using ~ 5 million genome-wide markers to determine parental source populations worldwide [63]. The authors confirmed that ~ 95.3% of Afrikaner ancestry came from mostly northwestern European populations, with the remaining section contributed by admixture with slaves and the local Khoe-San groups [63].

The absence of CNVs in this group could be due to a small sample size (*n* = 110), incorrect self-identification due to a lack of knowledge regarding family history/ancestry of the English speaking non-Afrikaner patients, or the fact that potential European ancestors carrying these CNVs did not contribute to the overall mutation spectrum in this group.

The contribution of LGRs in the SA population could change in the future. Of the LGRs reported, the majority (3.5%) were identified in English-speaking families with evidence of a Western/Northern European heritage (Table 1), with none detected for the Afrikaner [12] and a single case reported for the Black SA population (*BRCA1* exon 21 presented in the current study) (Table 1). Together, these two groups account for 84.6% of the entire population, based on the 2011 SA census [64]. Therefore, all the SA LGRs were detected in patients identifying themselves as belonging to three minority groups that constitute only 15.2% of the total population [64].

## Conclusions

In summary, we report multiple new CNVs for the SA population, ranging from single exon deletions or duplications to WGDs. This paper is the first to described LGRs identified for representative SA ethnicities such as the Coloured, SA Indian and Black populations. Larger genomic rearrangements do contribute to familial BC and OVC in SA, with a contribution of 8.7% to the overall mutational burden of *BRCA1/2*. These LGRs are currently mostly restricted to three minority SA population groups, with the majority identified for patients linked to a Western/Northern European heritage (White non-Afrikaner). The complete deletion of *BRCA2* is, however, a rare finding.

## Data Availability

The datasets generated and/or analysed during the current study are available in the Leiden Open Variation Database (LOVD^3^; user account #03562), repository https://urldefense.proofpoint.com/v2/url?u=https-3A__databases.lovd.nl_shared_users_03562&d=DwIBaQ&c=vTCSeBKl9YZZHWJzz-zQUQ&r=hcehu07Ya-T_uQsJJSbMASuJgO-QMsJlTaPwnW9uPQ8&m=EWkSAimPuSdE1W-WAqT0RuX2un0j0L2GSzRJfvrsLTk&s=4t5Ghmkjq6BVeInYjj3oyutXdIj4UXrMDtgGet8XVWg&e=.

## Abbreviations

BC: Breast cancer
bp: base pair
CNV: Copy number variation
EDTA: Ethylenediamine tetraacetic acid
kb: kilobase
MLPA: Multiplex ligation-dependent probe amplification
NGS: New Generation Sequencing
OVC: Ovarian cancer
SA: South Africa
SNP: Single nucleotide polymorphism
SNV: Single nucleotide variant
WGD: Whole gene deletion
